# Hemoperfusion: technical aspects and state of the art

**DOI:** 10.1186/s13054-022-04009-w

**Published:** 2022-05-12

**Authors:** Claudio Ronco, Rinaldo Bellomo

**Affiliations:** 1 Department of Medicine, University of Padova, Padua, Italy; 2 International Renal Research Institute of Vicenza (IRRV), Vicenza, Italy; 3 Department of Nephrology, San Bortolo Hospital, Vicenza, Italy; 4 Department of Critical Care, University of Melbourne, Melbourne, Australia; 5 Australian and New Zealand Intensive Care Research Centre, Monash University, Melbourne, Australia; 6 Data Analytics Research and Evaluation Centre, Austin Hospital, Melbourne, Australia; 7 Department of Intensive Care, Austin Hospital, Heidelberg, Melbourne, VIC 3084 Australia; 8 Department of Intensive Care, Royal Melbourne Hospital, Melbourne, Australia

## Abstract

**Background:**

Blood purification through the removal of plasma solutes by adsorption to beads of charcoal or resins contained in a cartridge (hemoperfusion) has a long and imperfect history. Developments in production and coating technology, however, have recently increased the biocompatibility of sorbents and have spurred renewed interest in hemoperfusion.

**Methods:**

We performed a narrative assessment of the literature with focus on the technology, characteristics, and principles of hemoperfusion. We assessed publications in ex vivo, animal, and human studies. We synthesized such literature in a technical and state-of-the-art summary.

**Results:**

Early hemoperfusion studies were hampered by bioincompatibility. Recent technology, however, has improved its safety. Hemoperfusion has been used with positive effects in chronic dialysis and chronic liver disease. It has also demonstrated extraction of a variety of toxins and drugs during episodes of overdose. Trials with endotoxin binding polymyxin B have shown mixed results in septic shock and are under active investigation. The role of non-selective hemoperfusion in sepsis or inflammation remains. Although new technologies have made sorbents more biocompatible, the research agenda in the field remains vast.

**Conclusion:**

New sorbents markedly differ from those used in the past because of greater biocompatibility and safety. Initial studies of novel sorbent-based hemoperfusion show some promise in specific chronic conditions and some acute states. Systematic studies of novel sorbent-based hemoperfusion are now both necessary and justified.

## Introduction

The removal on unwanted plasma solutes by direct adsorption has an established long history. However, early sorbent technology had major bioincompatibility problems (e.g., thrombocytopenia, leukopenia, hypoglycemia, hypocalcemia). This held back the development and clinical application of hemoperfusion. Sorbent biocompatibility, however, has improved triggering renewed interest, investigations, and application of hemoperfusion in clinical practice.

### Hemoperfusion: characteristics and principles

Extracorporeal blood purification can be achieved by different mass separation processes [1]. Diffusion, as in standard hemodialysis (HD), convection as in hemofiltration or their combination as in hemodiafiltration (HDF) [2]. While these techniques are based on membrane separation, a third mechanism, solute adsorption, is based on mass separation by a solid agent (sorbent) [3]. As current dialysis techniques present limitations due to membrane permeability characteristic, extracorporeal hemoperfusion represents an additional option for blood purification.

Sorbents have been studied for many years. Initially, inorganic aluminosilicates (zeolites) and charcoal were utilized for various purposes. In the last 50 years, however, organic polymer ion exchange resins and finally synthetic porous polymers (styrene or acrylic acid based) have been applied to blood purification (Table 1) [4]. Thus, while hemoperfusion techniques initially caused important adverse reactions and had problems with safe storage and priming, recent more biocompatible sorbent materials have been safely utilized for hemoadsorption techniques in various clinical settings.

**Table 1:** Development of sorbents and application in extracorporeal therapies

1850 First inorganic aluminosilicates (zeolites) used to exchange NH_4_ and Ca^++^
1910 Water softeners using zeolites display instability in the presence of mineral acids
1935 Adams and Holmes synthesize the first organic polymer ion exchange resin
1948 First published application of hemoperfusion using an ionic resin to treat uremia in dogs
1950s Application of synthetic porous polymers (trade names: Amberlyte, Duolite, Dowex) to experimental blood purification
1958. Use of ion exchange resin to treat a patient with barbiturate poisoning
1960s Clinical use of hemoperfusion with ion exchange resins to remove salicylate and phenobarbital in dogs
1970s Widespread application of coated charcoal and resins to the treatment of poisoning
1980s Application of coated charcoal and resins to the treatment of a variety of conditions (liver disease, vasculitis, and autoimmune diseases)
1990s Decreased interest in hemoperfusion with charcoal and resins and side effects reported more frequently with greater use
2000s Continued decrease in the use of hemoperfusion as dialysis membranes achieve better clearance, greater biocompatibility and lower cost and continuous renal replacement therapy spreads
2010s Improvements in coating and manufacturing and positive experimental work restore interest in hemoperfusion with growing numbers of reports
2020s Application of hemoperfusion to the management if inflammatory and/or septic states becomes more common

Sorbents have a very large surface/volume ratio and a significant capacity to bind specific solutes thanks to weak ionic bonds, Wan der Waals forces, and strong hydrophobic bonds. They can be natural raw materials, or be synthetically produced [5]. Zeolites (alumina-silicates) are inorganic porous polymers with a level of porosity, derived from their crystal structure (today synthetically modulated to control the structure of the internal pore system). Porous carbons, instead, are cellulose-derived organic polymers prepared by controlled thermal oxidation. Finally, almost all polymerizable monomers can be built up into large molecules via a multitude of reactions, using divinylbenzene as a potent crosslinking substance. Monomers can be bi-functional (creating linear polymers) or multifunctional (creating a cross-linked network polymer structure). The latter is the intrinsic nature of the most recent synthetic sorbents that can be further functionalized by surface-coating with biocompatible polysulfone [6].

Today, adverse plasma-to-sorbent-induced reactions have become uncommon and can be prevented by plasma separation prior to circulation through the sorbent bed. After the sorbent cartridge, blood is reconstituted so that red cells, white cells, and platelets never meet the sorbent surface, and bio-incompatibility reactions are avoided [7]. Alternatively, the sorbent is made biocompatible by a specific coating process covering the particles with bio-layers that are well tolerated by blood cells [8].

Sorbents are generally produced in granules, beads, or fibers. They are solid particles with a diameter generally ranging between 50 µm and 1.2 cm. The surface-area-to-volume ratio (S/V) is extremely high with a surface area varying from 300 to 1200 m^2^/g. In addition, sorbents are classified according to the size of the pores of their inner structure as a) Macro-porous (Pore size > 500 Å), b) Meso-porous (Pore size 20–500 Å) and c) Micro-porous (Pore size < 20 Å). The requirements for an ideal sorbent material are reported in Table 2 [9].

**Table 2:** Requirements for ideal sorbent material and optimal cartridge design

**Sorbent material**
High selectivity/affinity to enable sharp separation
High capacity to minimize the amount of sorbent needed
Favorable kinetic and transport properties for rapid sorption
Chemical and thermal stability; low solubility when contacting fluid
Hardness and mechanical strength to prevent crushing and erosion
Free flowing tendency for easy filling and emptying of the packed beads
High resistance to fouling for long life and low solute interference
No tendency to promote undesirable chemical reactions or side effects
Relatively low cost
**Sorbent cartridge**
Adequate design in terms of length and diameter
Adequate internal volume to avoid excessive blood priming volume
Avoidance of dead space zones where easy clotting may occur
Adequate packing density of the sorbent particles
Low resistance to blood flow of the packed bed
Adequate retention screens at the ports to avoid sorbent particles dissemination
Mass transfer zone shorter than unit length

Once the sorbent particles are produced, their packing into a device (cartridge) requires a tortuous pathway (sorbent bed) through which blood or any fluid phase must flow. Optimal packing density is generally between 35 and 55% of the available space. The optimal design of the cartridge depends on this factor and other factors listed in Table 2 [9].

When blood or plasma is circulated through the sorbent bed, removal of solutes by adsorption takes place on the surface of the beads. Maximum adsorption is achieved when equilibrium is reached, i.e., when the concentration of the marker solute at the outlet of the unit equals the concentration at the inlet. No theory for predicting adsorption curves has been universally embraced. Instead, laboratory experiments must be performed at fixed temperature (separation processes are energy intensive and affect entropy) for each liquid mixture and sorbent to provide sufficient data to derive specific plotting curves called *adsorption isotherms*.

At equilibrium, the following equation applies:

$$C_{{{\text{b}}({\text{initial}})}} \times V_{{\text{b}}} = \, \left( {C_{{{\text{b}}({\text{final}})}} \times V_{{\text{b}}} } \right) + \left( {C_{{\text{s}}} \times S} \right)$$

where *C*_b(initial)_ is the concentration of the solute at the beginning of the experiment; *V*_b_ is the total volume of the carrier fluid (constant during the experiment), *C*_b(final)_ is the concentration of the solute at the end of the experiment (when equilibrium takes place), *C*_s_ is the concentration of the adsorbate (mol/unit of sorbent mass), and S is the total mass of the sorbent available for mass transport.

Adsorption isotherms can be used to determine the amount of sorbent required to remove a given amount of solute. However, isotherms may differ with different unit design. This depends on the packing density of the sorbent, the length and inner diameter of the unit (cartridge), and the inter-particle distance and path tortuosity, all of which regulate the flow dynamics inside the unit. The flow characteristics through a sorbent bed are also governed by physical laws such as Darcy’s law and the Kozeny–Carman equation, which is used to calculate the pressure drop for a fluid flowing through a packed bed of solids. However, discussion of these additional laws and equations goes beyond the scope of this manuscript.

In practice, the adequacy of unit design can be evaluated by measuring its solute mass transfer zone (MTZ). The MTZ (expressed in cm) is represented by the distance between the point (cross section) where all sorbent material is saturated, and the point where zero adsorbate is present in the sorbent. Depending on flow distribution inside the unit, the MTZ may be very short (less than 1/3 of the unit length), equal to or longer than unit length. In the last two cases, a flow-through condition is experienced (i.e., a condition when solute is present at the outlet of the unit, this leaving behind some unused sorbent mass) [10].

The main goal of constructing an optimal sorbent cartridge is to obtain the maximum contact of the fluid phase with the entire amount of available sorbent. There are various steps, however, in such adsorption process:

- External (interphase) mass transfer of the solute from the bulk fluid by convection through a thin film or boundary layer to the outer surface of the sorbent;
- internal (intra-phase) mass transfer of the solute by pore diffusion from the outer surface of the adsorbent to the inner surface of the internal porous structure;
- surface diffusion along the porous surface and
- adsorption of the solute onto the porous surface.

During clinical use, the final kinetics also depend on the extracorporeal blood flow and the initial concentration of the marker molecule. These factors may result in earlier saturation or prolonged efficiency of the hemoperfusion unit [11].

## The logic behind hemoperfusion

### Why remove solutes directly from blood with sorbents

The concept that some disease states are associated with the presence of injurious molecules (solutes) in blood is well-established and the basis of life-saving treatments like dialysis. Some solutes are very large and can only be removed by plasma exchange. However, other toxic solutes are small enough to be removed by dialysis or by adsorption to sorbents beads [12]. Such coated sorbent beads can be packed into a cartridge to enable inclusion into an extracorporeal circuit. This process allows the contact of blood with the sorbent for a sufficient period to allow removal of target solutes with limited activation of the immune system [3]. This approach is attractive because it is direct, technically relatively simple, and theoretically efficient [13].

### The plasma exchange or plasmafiltration option

Plasma exchange or plasma filtration [14] are techniques that enable the removal of most molecules present in plasma, spanning in size from any small solute to large protein such as globulins [15]. These techniques remove a broad array of molecules that are believed to be toxic as in sepsis [16] or severe liver failure [17]. However, removal is non-selective and simultaneous removal of a large array of potentially beneficial or necessary molecules (clotting factors, albumin, antibiotics, and protective antibodies) takes place. Consequently, replacement of such losses requires the administration of albumin and/or fresh frozen plasma, with the problem of cost and blood product consumption. Moreover, as therapy continues or becomes more intensive, it has the effect of removing the very “non-toxic” plasma administered to cover the losses of clotting factors. Thus, outside of specific situations [15], plasma exchange has not achieved widespread application.

### The rationale for blood purification in “toxic states”

When key homeostatic organs (e.g., kidneys, liver, and immune system) malfunction, toxic solutes accumulate [18]. This provides the rationale for blood purification therapy. In the case of dialysis, this rationale has led to a life-saving therapy for millions of patients over the last 50 years. For liver failure or immune dysfunction; however, no equivalent therapy has yet been developed.

Nonetheless, the concept of blood purification therapy is supported by multiple ex vivo studies [19–21] and experimental animal studies [22, 23]. All show that a wide array of endogenous and exogenous toxins (including endotoxin, poisons and drugs) can be removed by blood purification techniques [24–26]. Such studies have also shown clinical and survival benefit in animal models. However, the efficiency of toxin removal with current systems may be inadequate in human disease [27] and animal studies do not offer a robust prediction of clinical effect.

### Removal of protective solutes

A logical concern with blood purification by any technique that is not highly specific is that it will lead remove protective solutes (e.g., antibiotics or anti-inflammatory substances, protective cytokines, amino acids, macro- and micronutrients and other circulating potentially protective metabolites). Such removal might be as quantitatively important as the removal of toxins. However, in predominantly toxic states, the dominant view is that the accumulation of toxins likely outweighs that of protective molecules. Thus, any broad removal technique will remove more toxic than protective molecules [28]. It remains unclear whether this paradigm is true or not. Such uncertainty stems from the fact that we have a very limited understanding of such protective molecules. Thus, in sepsis, the only molecules we currently understand to be protective are antimicrobial drugs. However, while extensive clearance data exists for different forms of renal replacement therapy [29], the data for antibiotics and antifungal drugs removal during hemoperfusion is scant or absent.

### Selective vs non-selective hemoperfusion in sepsis

Blood purification in sepsis has been a key area of investigation because of the view that soluble mediators of injury are a major contributor to morbidity and mortality in septic patients [30]. Such mediators appear to span a wide array of molecular size and are potentially amenable to removal by hemoperfusion. In the field of hemoperfusion in sepsis, two approaches have been developed, one based on selective targeting of a key molecule (e.g., endotoxin) and the other based on non-selective adsorption.

The concept of endotoxin adsorption has been based on several trials of the endotoxin-binding ability of polymyxin B as discussed in detail below [31].

The effectiveness of the broad adsorption strategy for sepsis, on the other hand, has not yet been tested in suitably designed multicenter randomized controlled trials. Thus, it lacks experimental and clinical robustness.

Nonetheless, two sorbent technologies have emerged: the Cytosorb cartridges [32, 33] and the Jafron HA cartridges series [34]. These sorbents have been used as rescue therapy in sepsis or as adjuvant therapy in sepsis [34] and experience has accumulated in terms of technique and safety [35]. However, that before substantial randomized controlled studies are designed and performed, more work is needed regarding what technical parameters (e.g., blood flow, cartridge size, length and composition and duration of use) define the optimal operative characteristics of such technology.

## Technical aspects of hemoperfusion

The extracorporeal circuit needed for hemoperfusion requires vascular access with a double lumen catheter placed in a central vein. Hemoperfusion, however, can also be applied to the treatment of chronic patients and in combination with hemodialysis [36, 37] via arterio-venous fistulas. The extracorporeal circuit requires a hemodialysis or a continuous renal replacement therapy (CRRT) machine, or, in some cases, a simple blood pump with pressure alarms. Depending on the indications, the characteristics of the patient, the duration of the session, and the technique utilized, anticoagulation of the extracorporeal circuit can be optimized. In some patients, regional citrate anticoagulation can be employed, while in some others at high hemorrhagic risk, treatment can be performed without anticoagulation.

Due to the nature of the sorbent cartridges, the extracorporeal circuit may undergo modifications leading to different techniques.

**Fig. 1:**
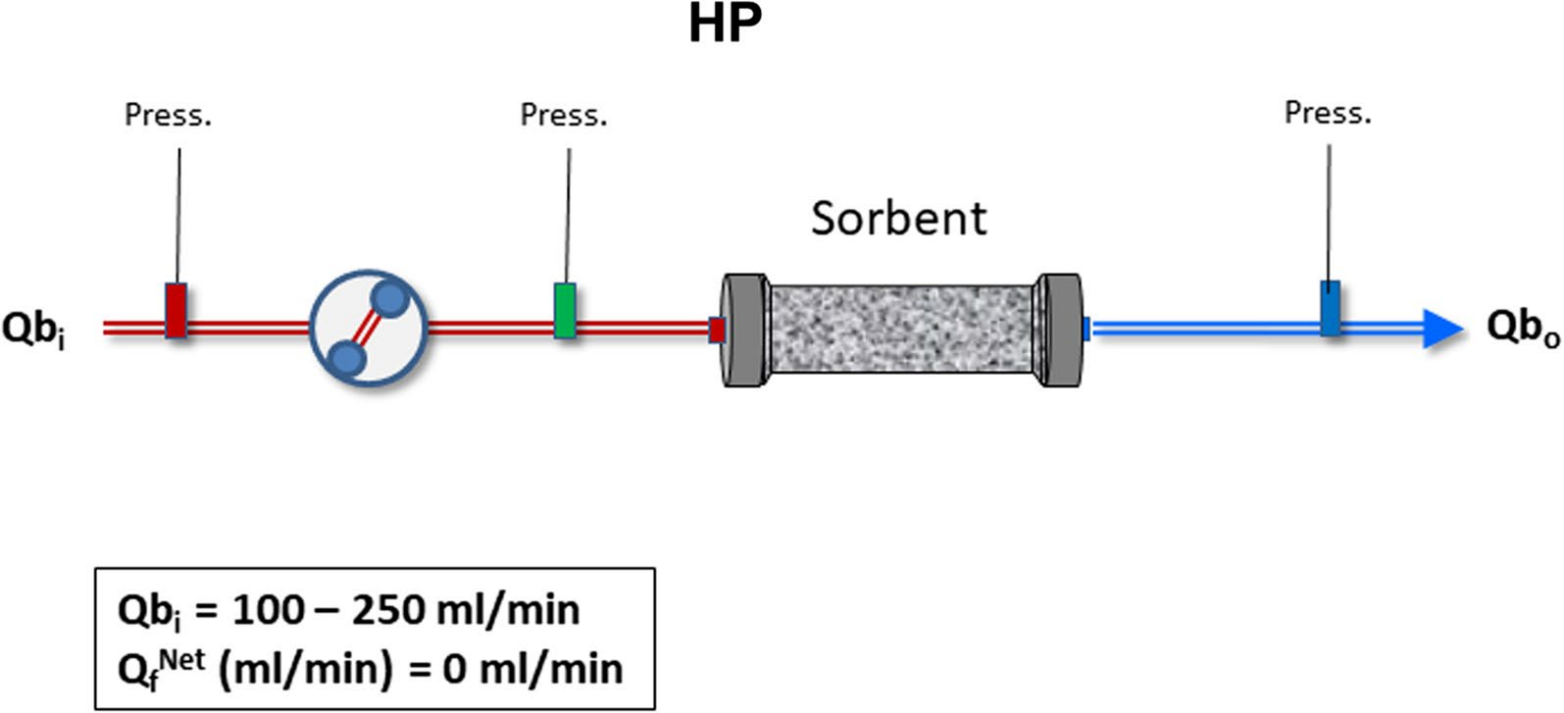
Schematic configuration of direct hemoperfusion (HP). *Q*b_i_ = Blood flow at the inlet of the unit; *Q*_f_^Net^ = net ultrafiltration

*Hemoperfusion (direct hemoadsorption) (HP):* Blood is circulated by a pump through a sorbent unit (cartridge) and enters in direct contact with the sorbent particles [38] (Fig. 1). Blood flow may vary according to the size of the cartridge (100–250 ml/min). The extracorporeal circuit is anticoagulated with heparin or citrate.

**Fig. 2:**
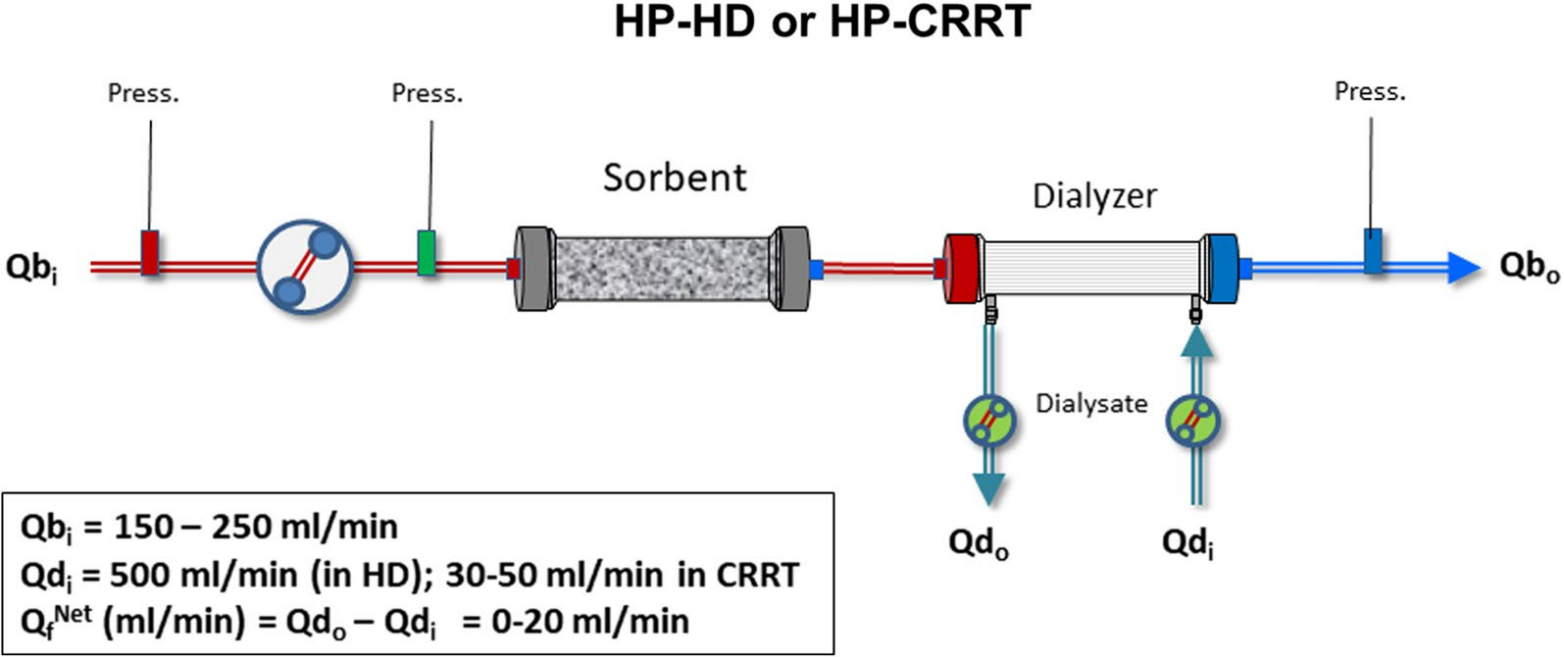
Schematic configuration of hemoperfusion combined with hemodialysis (HP-HD) and hemoperfusion combined with continuous renal replacement therapy (HP – CRRT). *Q*b_i_ = Blood flow at the inlet of the unit; *Q*b_o_ = Blood flow at the outlet of the units; *Q*d_i_ = Dialysate flow at the inlet of the dialyzer; *Q*d_o_ = Dialysate flow at the outlet of the dialyzer; *Q*_f_^Net^ = net ultrafiltration

*Hemoperfusion combined with dialysis/CRRT*: The sorbent is utilized in combination with hemodialysis (HP-HD) or with CRRT (HP-CRRT). As shown in Fig. 2, the sorbent can be placed before or after the dialyzer [39].

**Fig. 3:**
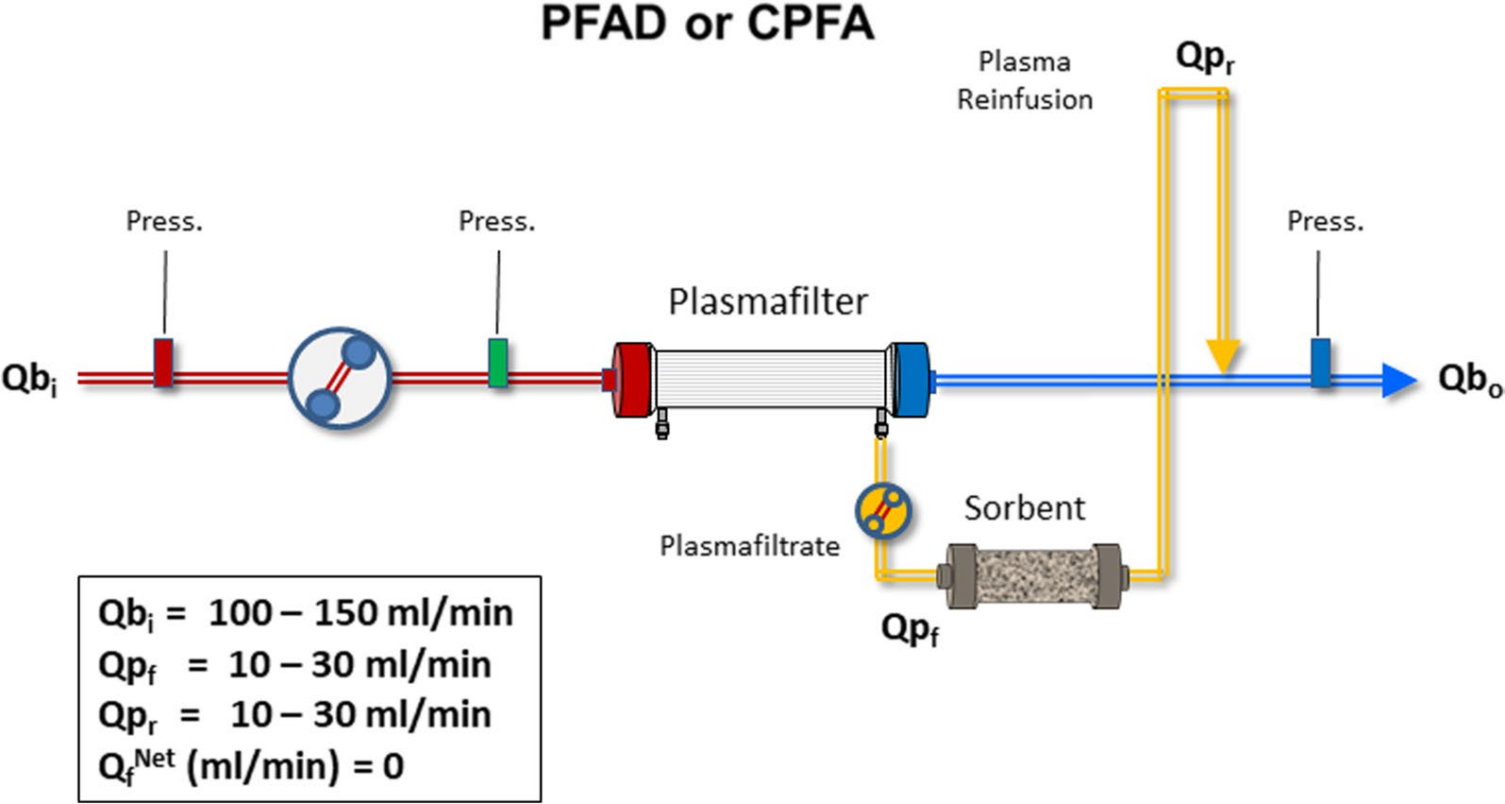
Schematic configuration of plasmafiltration-adsorption (PFAD) or continuous plasmafiltration-adsorption (CPFA). *Q*b_i_ = Blood flow at the inlet of the plasmafilter; *Q*b_o_ = Blood flow at the outlet of the plasmafilter; *Q*p_f_ = Plasmafiltrate flow; *Q*p_r_ = Plasma Reinfusion flow; *Q*_f_^Net^ = net ultrafiltration

*Plasmafiltration-adsorption*: Plasma is separated from blood, circulated through the sorbent and reinfused into the circuit. This technique can be performed for a few hours (PFAD = plasmafiltration-adsorption) or over a prolonged period (CPFA = continuous plasmafiltration adsorption) (Fig. 3) [40].

**Fig. 4:**
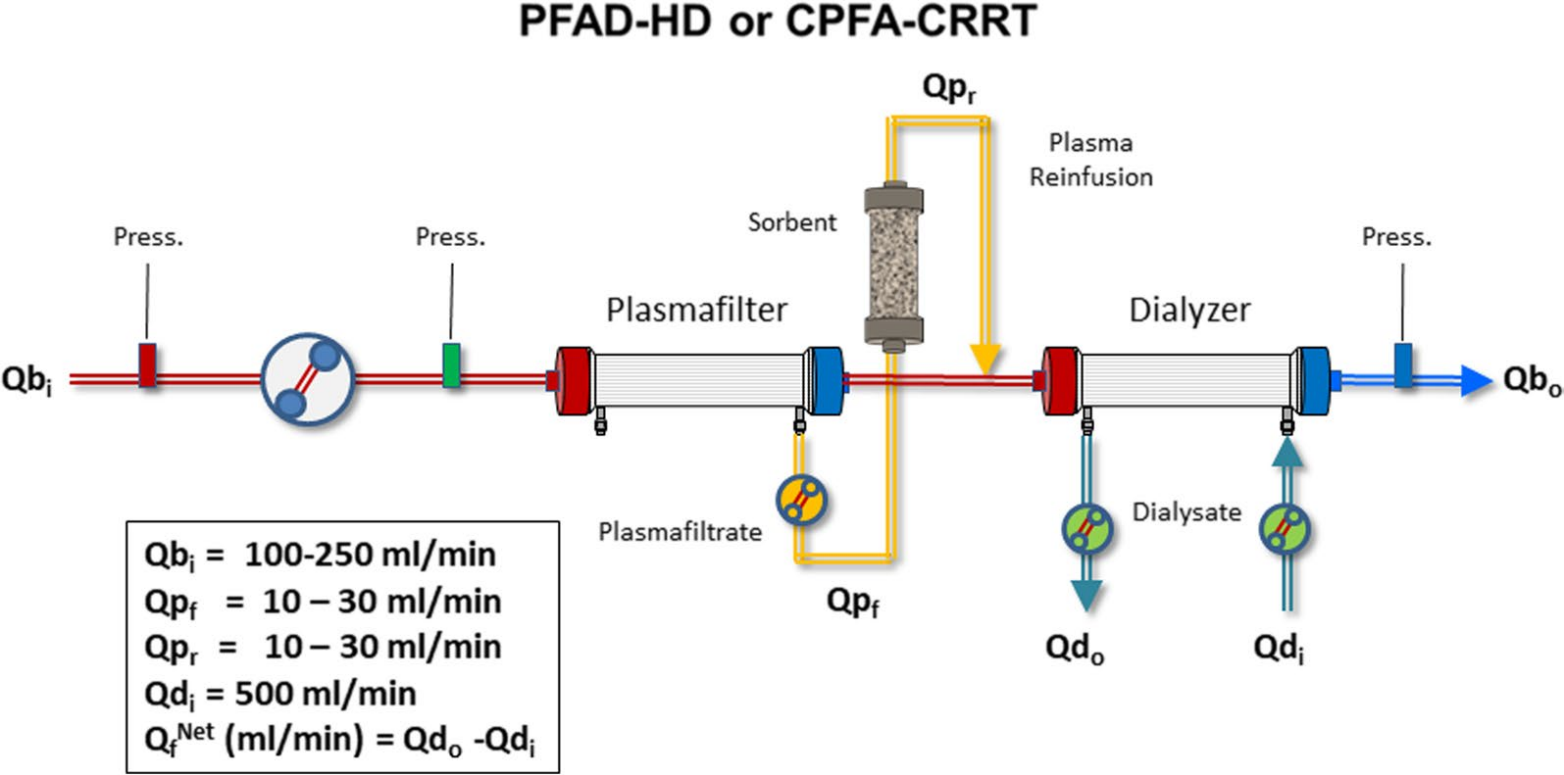
Schematic configuration of plasmafiltration-adsorption combined with hemodialysis (PFAD-HD) or continuous plasmafiltration-adsorption combined with continuous renal replacement therapy (CPFA-CRRT). *Q*b_i_ = Blood flow at the inlet of the units; *Q*b_o_ = Blood flow at the outlet of the units; *Q*p_f_ = Plasmafiltrate flow; *Q*p_r_ = Plasma Reinfusion flow; *Q*d_i_ = Dialysate flow at the inlet of the dialyzer; *Q*d_o_ = Dialysate flow at the outlet of the dialyzer; *Q*_f_^Net^ = net ultrafiltration

*Plasmafiltration-adsorption combined with dialysis/CRRT*: PFAD or CPFA can be combined with hemodialysis (PFAD-HD) or CRRT (CPFA-CRRT) to expand the efficiency of the treatment to small solutes such as urea and creatinine (Fig. 4) [16].

**Fig. 5:**
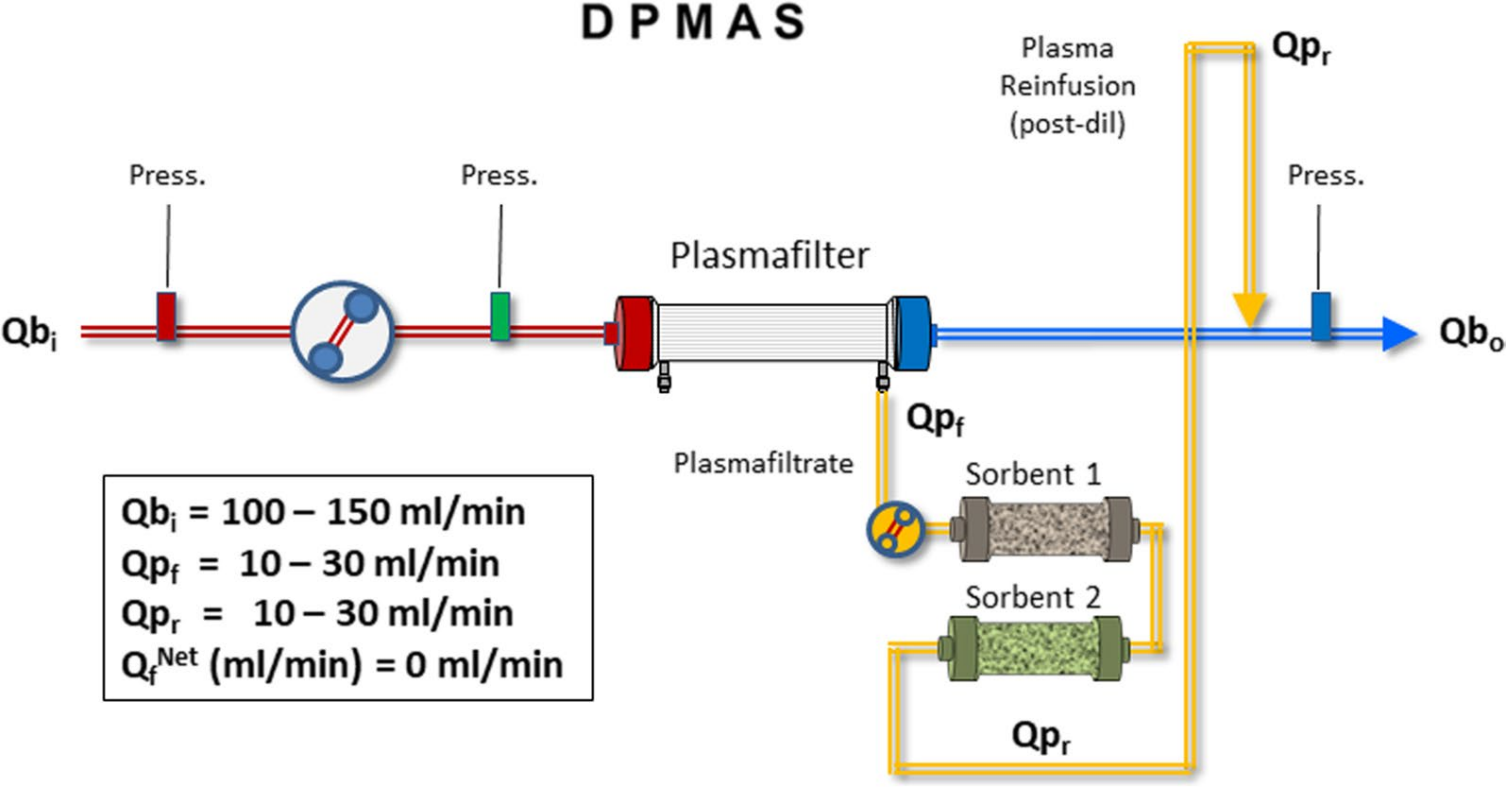
Schematic configuration of double plasmafiltration molecular adsorption system (DPMAS). *Q*b_i_ = Blood flow at the inlet of the unit; *Q*b_o_ = Blood flow at the outlet of the plasmafilter; *Q*p_f_ = Plasmafiltrate flow; *Q*p_r_ = Plasma Reinfusion flow; *Q*_f_^Net^ = net ultrafiltration

*Double plasmafiltration molecular adsorption system (DPMAS):* In some circumstances such as combined kidney and liver failure, different sorbent units with specific characteristics can be placed in the plasmafiltration circuit (Fig. 5). The nature of the sorbents and the characteristics of the cartridges depend on the indications and the degree of severity [41]

**Fig. 6:**
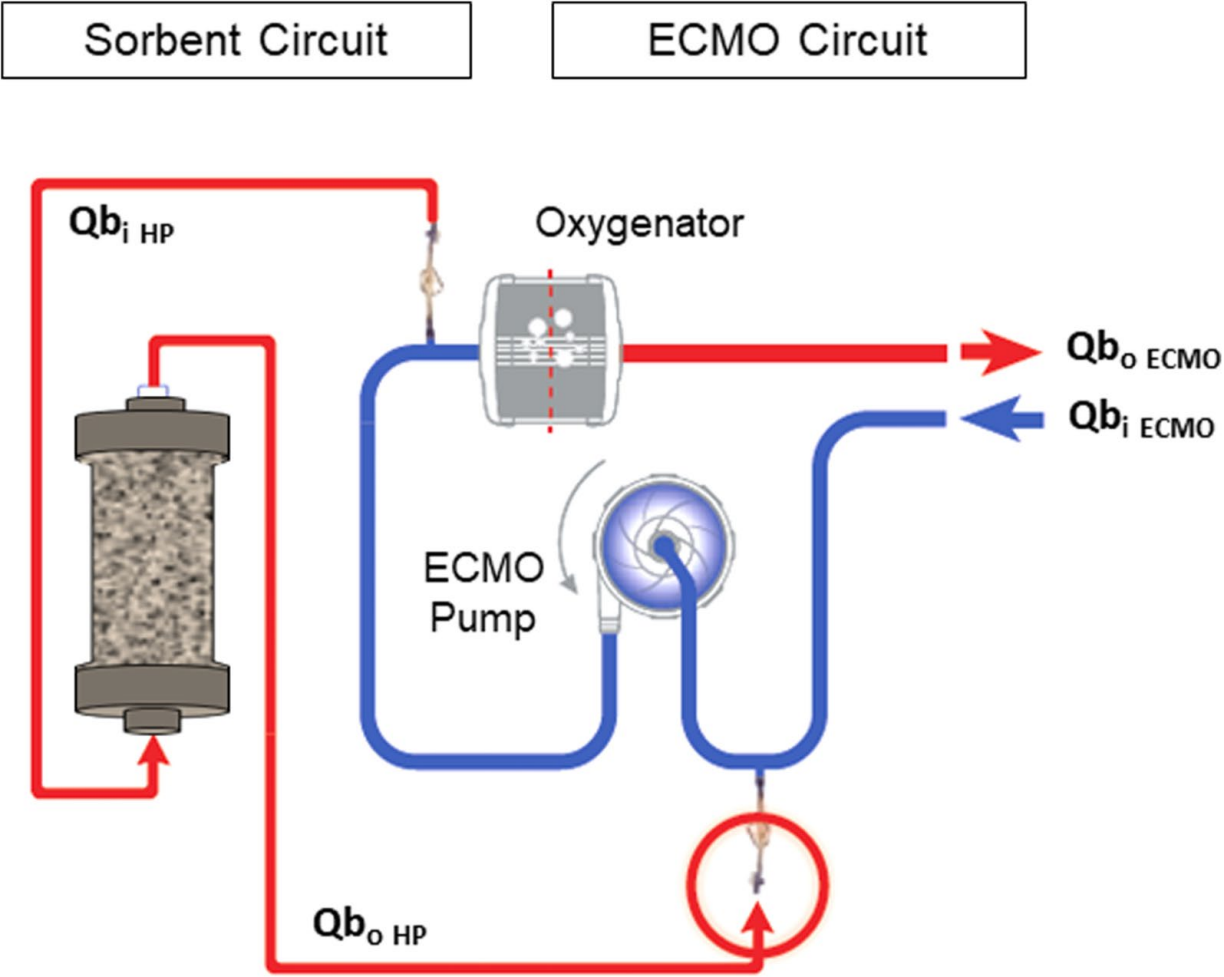
Schematic configuration of direct hemoperfusion combined with extracorporeal membrane oxygenation (HP-ECMO). *Q*b_i HP_ = Blood flow at the inlet of the hemoperfusion unit; *Q*b_o HP_ = Blood flow at the outlet of the hemoperfusion unit; *Q*b_i ECMO_ = Blood flow at the inlet of the ECMO circuit; *Q*b_o ECMO_ = Blood flow at the outlet of the ECMO circuit

*Hemoperfusion in combination with ECMO*: In patients undergoing veno-venous or veno-arterial extracorporeal membrane oxygenation (VV-ECMO or VA-ECMO) hemoperfusion may be connected to the ECMO circuit. However, the sorbent and pressure gradients should be adjusted to achieve adequate flows while avoiding perturbations of the main circuit [42] (Fig. 6). Similar circuits can be created during cardiopulmonary bypass.

All these approaches have been utilized with technical success and no major adverse events. However, several aspects still require technical and clinical studies. First, it is necessary to define solute kinetics and isotherms for specific solutes and different devices. Second, more work is needed to define the optimal duration of treatment in relation to blood blow, cartridge saturation, and clotting risk. Third, in the clinical environment, we need to correctly phenotype the patient, to identify criteria for initiation and cessation of hemoperfusion, to define optimal “adsorption dose” for a given patient and finally to identify marker molecules and clinical parameters to characterize the efficacy of the therapy and help design future trials. In their absence, the decision to use hemoperfusion remains based on clinical judgement and local expertise.

## When to consider hemoperfusion

There are no established indications for hemoperfusion. However, several biologically and pathophysiological rational indications have emerged.

### Intoxication

Several indications have been explored in relation to hemoperfusion in the setting of “intoxication.” As an example, they might include the treatment of intoxication with either a drug [43–45] (e.g., valproate, carbamazepine) or a toxic chemical (e.g., paraquat or organophosphates) [46, 47] or toxic natural products [48] (e.g., mushroom-related toxins). Unfortunately, no controlled studies exist. What is available, however, is information on extraction rate, clearance, and mass removal. Over the last decade, the hemoperfusion devices most commonly used for such treatment have been commercial Cytosorb® cartridges or the HA Jafron Biomedical series, with extraction rates from 20 to 90% [12].

### Liver disease

There is very limited information or research in the use of hemoperfusion for severe liver failure (either acute or acute on chronic) even though there is a robust rationale for targeting ammonia or bilirubin in this setting [49]. However, there may be a role of hemoperfusion for the treatment of intractable cholestatic pruritus [49]. The available evidence is based on no controlled clinical trials and depends on sporadic case reports or small case series, thus preventing any conclusions [50].

### Renal disease

A variety of end-stage renal failure-associated toxins are not adequately removed during dialysis justifying the combined use of resins in selected patients to address issues such as beta-2 microglobulin removal or uremic pruritus Initial reports are encouraging [36, 51].

### Sepsis

#### Selective hemoperfusion

Several trials have addressed the possible effectiveness of hemoperfusion with polymyxin-bound membranes for the removal of endotoxin in patients with sepsis. These studies have consistently used the Toraymyxin™ (Toray Medical Co.Ltd., Tokyo, Japan) cartridge [52].

The first randomized trial (EUPHAS) was reported in 2009 and involved 64 patients with septic shock due to an abdominal cause. It randomized 34 patients to polymyxin (PMX) B hemoperfusion and 30 to conventional care [53]. EUPHAS reported physiological advantages on blood pressure, gas exchange, and vasopressor use with PMX but no change in the control population. In addition, PMX decreased time to mortality.

The second study was a multicenter randomized controlled trial of early PMX hemoperfusion in septic shock due to peritonitis [54]. It randomized 125 patients to PMX and 118 to conventional treatment. It found no benefit and a trend toward earlier time to mortality with PMX.

The third study was the EUPHRATES trial [55]. This trial compared PMX hemoperfusion to conventional therapy in 450 critically ill patients with septic shock and an endotoxin assay activity of ≥ 0.60 in 55 North American hospitals. This trial found no survival advantage among all participants or in the pre-specified subgroup with a multiorgan dysfunction score > 9. However, a post hoc assessment of patients without extreme endotoxemia [56] found a survival advantage on time to event analysis.

Finally, a new study called TIGRIS is currently under way in patients with endotoxemic septic shock (Clinical Trials.gov identifier: NCT03901807). This is a 150-patient prospective, multicenter, randomized, open-label trial of standard medical care plus the PMX versus standard medical care alone, for the treatment of subjects with endotoxemia (endotoxin activity ≥ 0.60 and < 0.90) and septic shock.

#### Non-selective hemoperfusion

Hemoperfusion with the CytoSorb® cartridge (CytoSorb®, Cytosorbents Inc, New Jersey, USA) represents a form of generic anti-inflammatory strategy and has been studied in case series and small comparative studies [57–61].

A multicenter randomized trial compared Cytosorb® therapy with conventional care [62] in 100 ventilated patients with sepsis or septic shock and either acute lung injury or ARDS. The primary outcome was the change in normalized interleukin-6 (IL-6) during study day 1 to 7. Although the CytoSorb® device had a single pass IL-6 extraction of between 5 and 18%, there were no significant differences in IL-6 levels.

More recently, CytoSorb®-based therapy was tested in COVID-19 patients on veno-venous extracorporeal membrane oxygenation (ECMO) [63]. In a single-center, open-label, randomized trial, 17 ECMO patients were treated with CytoSorb® for 72 h and 17 without. The decrease in IL-6 levels was similar but survival after 30 days was 18% with CytoSorb® therapy and 76% without (p = 0.0016). These mortality findings may represent a type 1 error in a small trial but raise concerns about the safety of this treatment in the setting of ECMO therapy.

Hemoperfusion with the JAFRON HA cartridge series (Jafron Biomedical, Guangdong, China) has been used in sepsis and reported in case series [64, 65]. One non-randomized study [66] involved 24 treated patients and 20 controls. It reported hemodynamic benefits, reduced interleukin 8 and 6 levels, and beneficial effects on ICU length of stay but no significant effect on mortality (46% in treated patients vs. 55% in control patients). Another study randomized 46 patients with acute lung injury and sepsis to daily treatment with the HA330 Jafron cartridge for three days vs usual care. HA-330 decreased TNF and IL-1 levels, improved markers of lung injury, duration of mechanical ventilation, CRRT and even 28-day mortality (67% in treated patients vs. 28% in control patients) [67]. In a third randomized trial of 30 patients, hemoperfusion with the same cartridge once a day was combined with pulse high-volume hemofiltration and was associated with beneficial effects on cytokines and cardiovascular physiology but no effect on mortality [68].

More recently, hemoperfusion with the Seraph® 100 Microbind® Affinity Blood Filter (ExThera, Martinez, CA) was approved by the FDA under Emergency Use Authorization for the treatment of severe COVID-19 [69]. This device contains adsorptive beads of ultrahigh molecular weight which, in vitro, remove the COVID-19 virus [70]. However, no randomized trials have yet been published [71].

Two randomized controlled studies of Cytosorb® have been published in 2022. The first studied hemoperfusion during cardiac surgery for infective endocarditis, with the device integrated into the cardiopulmonary bypass circuit [72]. It randomized 142 patients to Cytosorb® and 146 to usual care and found no differences in the primary outcome of change in SOFA score or in any other clinical outcomes, including mortality (21% vs. 22%). The second reported the effect of Cytosorb® therapy for 3 to 7 days in 50 COVID-19 patients with vasoplegic shock on time to resolution of shock [73]. It found no significant difference in this outcome and a mortality of 78% with Cytosorb® compared with 73% in the control arm.

Finally, in this review, we do not discuss the other techniques such as Molecular Adsorbent Recycling System (MARS) which provide a form of albumin adsorption treatment for liver disease. They do not represent direct hemoperfusion and are not strictly relevant to this review. Similarly, the use of hemofiltration membranes with greater adsorptive capacity (e.g., the oXiris membrane) does not constitute resin-based hemoperfusion and is also not directly relevant to this review.

## The hemoperfusion research agenda and recommendations

New sorbent materials has now paved the way for a resurgence of research into the clinical application of hemoperfusion [74].

Initial reports in the treatment of chronic dialysis patients and of chronic liver patients with pruritus have been encouraging [49, 75]. At the same time, clinical applications of sorbents in sepsis, acute kidney injury, and other inflammatory states have provided useful data on feasibility and safety, forming the basis for future technical, procedural, and manufacturing optimization [58, 76, 77].

Given such early data, and the lack of consensus clinical practice guidelines, we recommend that research should first focus on achieving a better understanding of the basic aspects of the adsorption process, the properties of each sorbent, the mechanisms of adsorption, and their potential side effects [78]. Second, we recommend that ex vivo studies targeting multiple relevant solutes (e.g., cytokines, ammonia, possible uremic toxins, toxic drugs, antibiotics) should be conducted to establish their ex vivo clearance with varying blood flow and duration of perfusion to define optimal operating conditions. Third, we recommend focusing research on identifying meaningful target molecules and measuring their intra-corporeal and extracorporeal kinetics. This could be done by utilizing the creation of isotherms and by studying changes in cartridge adsorption over time. Fourth, we recommend that studies with similar multiple target should be conducted in large animals to assess biological and physiological effects on organ function with prolonged exposure.

In clinical research, first we recommend focusing on identifying what conditions should trigger hemoperfusion treatment and for how long. In this regard, we recommend that a hemoperfusion registry be set up to collect data as in ECMO registries. Second, we recommend focusing on defining adequate, optimal and safe dosage of hemoperfusion for different disorders. We should study how best to measure efficiency and efficacy, how many sessions should be prescribed, and how often hemoperfusion cartridges should be changed. Thus, we recommend that all future human hemoperfusion studies should report on performance characteristics (e.g., clearance, excretion ratio, mass removal, performance for key biological targets). Finally, we recommend exploring the move from intermittent (short treatment of a few hours) to continuous (24/7) hemoperfusion therapy in high-risk states (e.g., sepsis, acute liver failure, severe intoxication). Finally, in key target diseases and once the above elements have been addressed, we recommend the development of programs of investigation based on randomization (from pilot feasibility studies, to phase II, and ultimately phase III studies where appropriate).

Unless these studies are conducted, the role of hemoperfusion will remain uncertain and inadequately understood. This will likely have adverse consequence on both patients and the development of the science of blood purification. In many ways, hemoperfusion is where continuous renal replacement therapy (CRRT) was at its inception in the 1970s and 1980s. Clinicians and nurses prescribing and delivering CRRT in 2022 would barely be able to recognize their modern CRRT in the first reports of continuous arteriovenous hemofiltration. It is quite possible that, with hemoperfusion, future clinicians will be in a similar position.

## Data Availability

Not applicable.
